# Physiological and performance parameters associated with critical power decline in hypoxia among highly-trained endurance athletes

**DOI:** 10.5114/biolsport.2026.153530

**Published:** 2025-08-29

**Authors:** Tomasz Kowalski, Adrian Wilk, Kinga Rębiś, Jadwiga Malczewska-Lenczowska, Andrzej Klusiewicz, Tadej Debevec, Raphael Faiss

**Affiliations:** 1 Department of Physiology, Institute of Sport—National Research Institute, Warsaw, Poland; 2 Department of Nutrition Physiology, Institute of Sport—National Research Institute, Warsaw, Poland; 3 Department of Physical Education and Health in Biala Podlaska, Faculty in Biala Podlaska, Jozef Pilsudski University of Physical Education, Warsaw, Poland; 4 Faculty of Sport, University of Ljubljana, Ljubljana, Slovenia; 5 Department of Automatics, Biocybernetics and Robotics, “Jozef Stefan” Institute, Ljubljana, Slovenia; 6 Institute of Sports Sciences, Faculty of Social and Political Sciences, University of Lausanne, Lausanne, Switzerland

**Keywords:** Cycling, Altitude, V̇O_2max_, Haematological status, Haemoglobin mass

## Abstract

We sought to investigate whether the magnitude of differences in cycling critical power between normoxia and hypoxia (∆CP) is associated with fitness level or haematological status in highly trained endurance athletes. Thirty-three triathletes and longtrack speed skaters (11 females) completed two 3-minute CP cycling tests: one in normoxia (F_i_O_2_ = 20.8%) and the other in normobaric hypoxia (F_i_O_2_ = 14.2%). This cross-sectional study analysed ∆CP regarding performance, physiological, and haematological indices using correlation and regression analyses. Significant correlations were found between ∆CP and baseline CP in normoxia (r = -0.366, p = 0.047), ${\dot{\text{V}}\text{O}}_{2\max}$ (r = -0.437, p = 0.018), and MCH (r = 0.487, p = 0.012). Only a few significant associations were found between the indices obtained from venous blood sampling and ∆CP, different for females and males. In females, ∆CP was correlated with Hb_mass_ (r = -0.761, p = 0.017), erythrocyte volume (r = -0.783, p = 0.013), plasma volume (r = -0.745, p = 0.021), and blood volume (r = -0.870, p = 0.002), all established with the CO rebreathing method. The best-performing regression model (R^2^ = 0.501, RMSE = 0.033, p = 0.002, Cohen’s F^2^ = 1.004) included MCH, ${\dot{\text{V}}\text{O}}_{2\max}$, and Hb_mass_. A higher fitness level is associated with a greater CP decrease in hypoxia among the homogeneous cohort of highly trained endurance athletes. Haematological status plays a more prominent role in females, and the CO rebreathing method should be considered a preferred approach for assessing haematological status in highly trained athletes.

## INTRODUCTION

Exposure to hypoxia is associated with physiological adaptations that may significantly influence athletic performance. As hypoxia-related adaptations may, in the medium and long term, lead to improvements in performance and aerobic capacity, they are widely used in highperformance sports [1]. These adaptations tend to include haematological adaptations, such as an enhanced erythropoietic response resulting in increased haemoglobin mass (Hb_mass_), changes in mitochondrial gene expression, substrate utilization, hormone secretion, and improved muscle buffering capacity [2, 3]. Therefore, altitude training is often applied in endurance sports, with properly designed altitude interventions seen as a key factor in advancing future performance gains [4]. However, altitude exposure also leads to an ergolytic effect, typically impairing endurance performance due to reduced oxygen availability [5]. Although local oxygen diffusion capacity and extraction by active muscles are typically not impaired in hypoxia, the reduced oxygen uptake and transport still have an acute negative impact on endurance performance at altitude [6].

Moreover, training in hypoxia often results in reduced absolute intensities, while the relative intensity is adjusted to match sea-level equivalents. Such a reduction in absolute intensities usually reduces the mechanical stimuli, which may negatively impact training adaptations and subsequently performance [7, 8]. Moreover, multiple events in endurance sports are contested at altitude, including uphill finishes during cycling grand tours, trail running, cross-country skiing, or speed skating. Notably, even low altitude may negatively influence performance in trained endurance athletes [9]. Various strategies are constantly being explored to mitigate performance decline at altitude. Therefore, understanding factors associated with the magnitude of performance decrease at altitude remains highly important for athletes and coaches.

Several factors influencing performance at altitude have already been identified. They include acclimatization, cardiovascular function, training history, fitness level, muscle efficiency, buffering capacity, as well as nutritional and genetic features [2, 6, 10]. For example, endurance athletes exhibit a larger reduction in maximum oxygen uptake (${\dot{\text{V}}\text{O}}_{2\max}$) and oxygen delivery compared to the untrained population [11]. Obviously, detraining is not an optimal solution to mitigate the altitude-related performance decline in endurance athletes. Importantly, studies investigating the association between the degree of performance drop at altitude with fitness status typically are based on very distinct populations, such as active vs. sedentary or highly trained vs. untrained [12]. However, research addressing this relationship in homogeneous groups of highly trained athletes is lacking. Moreover, most studies focus on changes in ${\dot{\text{V}}\text{O}}_{2\max}$, not on changes in sport-specific performance. In our study, instead of ${\dot{\text{V}}\text{O}}_{2\max}$, we analysed critical power (CP), which is the highest sustainable power output one can maintain over a given period without losing homeostasis and which remains valid across different hypoxic conditions [13, 14]. The CP concept integrates metabolic, contractile, and respiratory systems, providing a framework exhibiting great scientific and practical utility [15].

Haematological status has been widely investigated in relation to altitude training, but mostly in regard to erythropoiesis and Hb_mass_ adaptations [16]. Simultaneously, multiple studies have reported that impaired haematological status, especially iron deficiency, typically hinders endurance performance [17]. Although the prevalence of iron deficiency anaemia and non-anaemia is noteworthy, most highly trained athletes do not exhibit haematological disorders [17]. However, the relationship between haematological variables within reference ranges and the extent of performance decline at altitude remains poorly understood and requires further investigation.

Therefore, the primary aim of this study was to examine whether the degree of difference in cycling CP between normoxia and hypoxia (∆CP) is associated with fitness level or haematological status. Importantly, the study investigates healthy and highly trained endurance athletes with confirmed haematological profiles. We hypothesized that a lower fitness level and superior haematological status within physiological reference ranges would be associated with a smaller decrease in CP at altitude.

## MATERIALS AND METHODS

### Study design

This is a cross-sectional study, based on data from purpose-applied CP testing and collected during routine assessments of elite athletes at the Institute of Sport – National Research Institute, Warsaw, Poland. The dataset used for the analysis was previously employed in a published study that addressed the transferability of muscle oxygen saturation across different ambient oxygen concentrations [18]. The study protocol was approved by the Ethics Committee of the Institute of Sport – National Research Institute, Warsaw, Poland (KEBN-24-97-KR). All participants provided written informed consent before taking part in the research. The study was conducted in compliance with the ethical principles outlined in the Declaration of Helsinki. The STROBE reporting guidelines for cross-sectional studies were applied [19].

The participants completed two 3-minute CP cycling tests in a randomized order with a day break: one in normoxia (87 m ASL, F_i_O_2_ = 20.8%) and the other in hypoxia (3200 m ASL, F_i_O_2_ = 14.2%). To maintain blinding, both tests took place inside a normobaric hypoxic chamber (Air Sport, Międzyzdroje, Poland). The placebo mode was used, and the athletes were not informed whether hypoxic conditions were applied. The operational noise, temperature (approximately 19°C), and humidity (around 50%) remained constant across the sessions. Before testing, they were already familiar with the CP protocol and were instructed to refrain from intense physical activity and long-distance travel for at least 48 hours. ∆CP was analysed in the context of CP levels, physiological indices (HR, ${\dot{\text{V}}\text{O}}_{2\max}$, SpO_2_, SmO_2_ in vastus lateralis, inspiratory muscle strength – IMS), and haematological variables (venous blood indices and multiple variables obtained by CO rebreathing method, including Hb_mass_). Cardiopulmonary testing and haematological status assessment were conducted across an 8-week span, from 28 days before to 28 days after CP cycling tests.

### Participants

Thirty-three highly trained endurance athletes (17 triathletes, 7 females; 16 speedskaters, 4 females) were included in the final analyses. They represented world-class or international level according to the Participant Classification Framework [20] and were recruited with convenience sampling. All the athletes were sea-level natives and residents. Inclusion criteria were: excellent health, all haematological variables within the reference ranges, relevant performance status in endurance sports, and experience with CP testing. Exclusion criteria were: any chronic or acute illness within the month before the first visit and during the whole assessment period, ongoing medication use, pregnancy, or exposure to hypoxia in the last six months. Additional exclusion criteria regarding inflammatory processes were applied during preliminary data screening, defined as: (1) c-reactive protein ≥ 10 mg · L^−1^ [21] or (2) erythrocyte sedimentation rate ≥ 20 mm · h^−1^ for females and ≥ 15 mm · h^−1^ for males [22]. The participants’ characteristics are presented in Table 1.

**TABLE 1:** The participants’ characteristics.

Variable/Group	Females (n = 11)	Males (n = 22)
Age [years]	21.3 ± 7.7	22.6 ± 6.2
BSA [m^2^]	1.64 ± 0.1	1.92 ± 0.12
CP [W/kg]	5.57 ± 0.8	5.92 ± 0.8
${\dot{\text{V}}\text{O}}_{2\max}$ [ml/kg/min]	56.2 ± 3.7	63.4 ± 4.6
Hb_mass_ [g/kg]	10.71 ± 0.65	12.48 ± 0.90
IMS [cmH_2_O/kg]	2.19 ± 0.37	2.19 ± 0.41

Values are mean ± standard deviation. Abbreviations: BSA – body surface area, CP – critical power (in normoxia), Hb_mass_ – hemoglobin mass, ${\dot{\text{V}}\text{O}}_{2\max}$ – maximum oxygen uptake, IMS – respiratory muscle strength

### Measurements and calculations

Body mass was measured with the InBody 770 (InBodyUSA, Cerritos, CA, USA) analyser. Body height was measured with the seca 274 (seca GmbH & Co. KG, Hamburg, Germany) free-standing digital stadiometer. Both measurements were performed at 8:00 AM, prior to breakfast. Body surface area (BSA) was calculated with the Mosteller formula [23].

Mechanical power was continuously measured throughout the 3-minute tests with the Wahoo KICKR trainer (Wahoo Fitness, Atlanta, GA) [24]. CP was established as the average from the last 30 seconds, separately for normoxia and hypoxia [25]. The difference in both variables between normoxia and hypoxia was calculated as a percentage (see below), and then transformed with the log_10_ approach [26]:

ΔCP=(CP in hypoxia−CP in normoxia)/CP in normoxia)×100

Systemic oxygen saturation (SpO_2_) was assessed at rest and after exercise with the NONIN Onyx Vantage 9590 finger pulse oximeter (Nonin Medical, Inc., Plymouth, Minnesota, USA). The resting measurements were taken after participants had remained seated for 30 minutes prior to each session (separate measurements were taken in normoxia and hypoxia). After the CP test, participants were immediately moved to the same seated position, and the subsequent measurement was taken one minute after exercise cessation. The difference in SpO_2_ (∆SpO_2_) between measures obtained before and after exercise was calculated as a percentage (see below), and then transformed with the log_10_ approach [26]:

$$\Delta {\text{SpO}}_{2}=({\text{SpO}}_{2}\text{ after exercise}-{\text{SpO}}_{2}\text{ before exercise})/{\text{SpO}}_{2}\text{ before exercise})\times 100$$

${\dot{\text{V}}\text{O}}_{2\max}$ was established as the highest 15-second average using Cortex Metamax 3B (Cortex Biophysik GmbH, Leipzig, Germany) and Cyclus II Ergometer (RBM, Leipzig, Germany) during a ramp test to exhaustion performed at sea-level (87 m ASL). The guidelines of the American College of Sports Medicine were applied for maximum effort criteria and ${\dot{\text{V}}\text{O}}_{2\max}$ determination [27]. Heart rate was monitored during the tests with a Polar H10 chest strap (Polar Electro Oy, Kempele, Finland). SmO_2_ was measured at rest and during exercise with a wearable near-infrared spectroscopy monitor named Moxy (Moxy Monitors, Hutchinson, MN, United States), mounted on the right vastus lateralis, approximately 13 ± 2 cm above the patella and 4 ± 2 cm lateral to the midline of the thigh. The monitor measures tissue oxygen saturation and presents arbitrary %SmO_2_ units on a 0–100% scale. The measurement spot was marked with a permanent marker to allow for consistent placement across sessions. To ensure proper shielding from ambient light, the Moxy device was covered using the manufacturer-provided light shield (128 × 115 mm) during all measurements. Physiotape, commonly used in kinesiology, was applied to prevent device displacement. Adipose tissue thickness was monitored at the marked spot and did not exceed a 15 mm threshold, as required for reliable measurements [28]. The difference in SmO_2_ in the vastus lateralis (∆SmO_2_) was calculated as a percentage (see below) and then transformed with the log_10_ approach [26]:

$$\Delta {\text{SmO}}_{2}=(\text{average }{\text{SmO}}_{2}\text{ during the last }30\text{ seconds of effort}-{\text{SmO}}_{2}\text{ before exercise})/{\text{SmO}}_{2}\text{ before exercise})\times 100$$

Critical oxygenation and critical heart rate were established as the averages from the last 30 seconds of the tests, separately for normoxia and hypoxia [18].

IMS was established with the S-Index Test, performed according to the guidelines from Kowalski and Klusiewicz [29].

The following indices were measured in whole blood using a haematology analyser (Sysmex XN-1000, Sysmex Corporation, Kobe, Japan): red blood cell count, white blood cell count, haematocrit, haemoglobin concentration, mean corpuscular volume of erythrocytes (MCV), and mean corpuscular haemoglobin (MCH), mean corpuscular haemoglobin concentration, reticulocyte haemoglobin equivalent (RET-HE). Erythrocyte sedimentation rate was established with VES-MATIC CUBE 30 (Diesse Diagnostica Senese S.p.A., Rigoni, Italy). C-reactive protein concentration, iron concentration, ferritin, soluble transferrin receptor, and total iron binding capacity were determined by the immunoturbidimetric method (Cobas Integra 400Plus, Roche Diagnostics, Mannheim, Germany). Blood samples were collected from the antecubital vein in the morning following an overnight fast, prior to any food intake. The procedure began after the participants had remained seated at rest for 15 minutes.

Hb_mass_ was determined using the CO rebreathing technique in duplicate. The rationale and the description of the applied procedures may be found in detail elsewhere [30]. The precision of Hb_mass_ assessment in our laboratory remains under the permissible 2% threshold. Intravascular volume indices, including plasma volume, blood volume, and erythrocyte volume, were additionally calculated.

All the measurements were conducted by experienced researchers and technicians, following scientific guidelines and the manufacturers’ instructions for the equipment. Variables were adjusted to body mass when necessary. Performance, oxygenation, and venous blood measurements were obtained for all the included participants (n = 33). IMS and measurements obtained by the CO rebreathing method were collected for 26 participants (13 triathletes, 7 females; 13 speedskaters, 2 females), and the missing values were not imputed. The study participants were familiar with the testing procedures and numerical fatigue assessment, as they had used them in training and testing many times before. All the testing procedures were conducted in July-August 2024 (preparatory period for speedskaters) and January-February 2025 (preparatory period for triathletes), in the laboratories of the Institute of Sport – National Research Institute (Warsaw, Poland).

### Statistical analysis

The data distribution was evaluated with the Shapiro-Wilk test and visual inspection of the plotted figures. Independent samples Student t-tests were performed to compare physiological and performance variables between males and females, and between triathletes and speedskaters. Pearson’s correlation analysis (combined and separately for females and males) and multiple linear regression (combined for females and males) were conducted to examine the relationship between ∆CP and included variables. Considering the sample size, no more than 3 predictors should be included in the regression analysis, as determined with G∗Power software (version 3.1.9.6, HHU, Germany). Therefore, after all the regression assumptions were tested, the best-performing model was established. Statistical significance was p ≤ 0.05. The statistical analyses were carried out with GraphPad Prism (version 10.4.1, GraphPad Software, USA).

### RESULTS

An exploratory analysis was performed to identify differences between the groups, correlations, and collinearity, and to guide further investigation. Significant differences in multiple physiological and performance variables were observed between males and females (p < 0.05), indicating potential limitations associated with the analysis of a combined sample. However, no significant differences were found between triathletes and speedskaters, either when analysed separately by sex or combined. The correlation analyses revealed several correlations between ∆CP and performance, physiological, and haematological variables.

In the combined sample, significant moderate correlations were found for ∆CP and baseline CP in normoxia (r = -0.366, p = 0.047), ${\dot{\text{V}}\text{O}}_{2\max}$ (r = -0.437, p = 0.018), and MCH (r = 0.487, p = 0.012).

In females, ∆CP was strongly correlated with CP in normoxia (r = -0.623, p = 0.041), critical heart rate in hypoxia (r = 0.636, p = 0.035), MCV (r = -0.671, p = 0.024), and RET-HE (r = -0.623, p = 0.041). Moreover, strong and very strong correlations were found between ∆CP and Hb_mass_ (r = -0.761, p = 0.017), erythrocyte volume (r = -0.783, p = 0.013), plasma volume (r = -0.714, p = 0.020), and blood volume (r = -0.870, p = 0.002).

In males, ∆CP was moderately correlated with haematocrit (r = -0.516, p = 0.041) and strongly correlated with MCH (r = 0.661, p = 0.005).

**FIG. 1:**
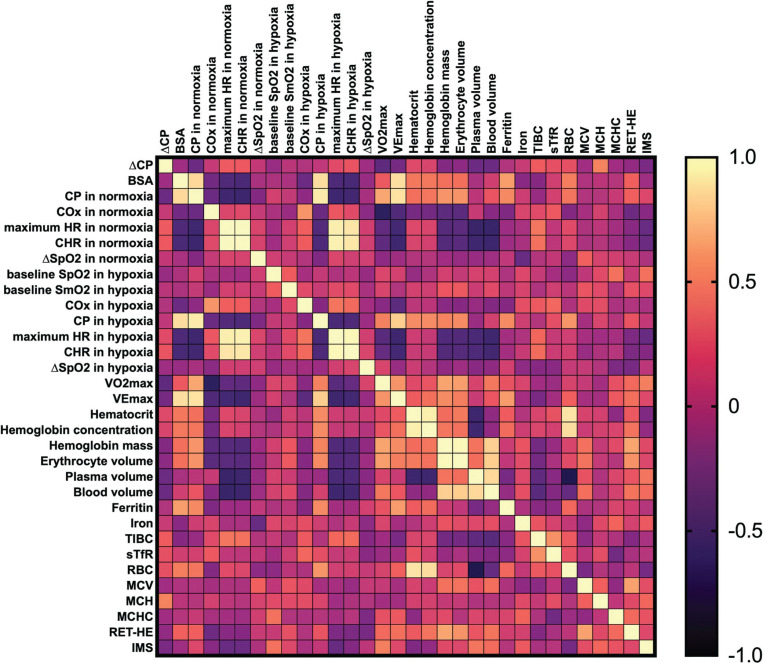
Pearson’s correlation matrix for key analysed variables in the combined sample. Abbreviations: ∆CP – differences in critical power between normoxia and hypoxia, BSA – body surface area, CP – critical power, COx – critical oxygenation, SpO_2_ – peripheral capillary oxygen saturation, SmO_2_ – muscle oxygen saturation in vastus lateralis, HR – heart rate, CHR – critical heart rate, ${\dot{\text{V}}\text{O}}_{2\max}$ – maximum oxygen uptake, VEmax – maximum ventilation, TIBC – total iron-binding capacity, sTfR – soluble transferrin receptor, RBC – red blood cell (count), MCV – mean corpuscular volume, MCH – mean corpuscular haemoglobin, MCHC – mean corpuscular haemoglobin concentration, RET-HE – reticulocyte haemoglobin equivalent, IMS – inspiratory muscle strength.

**FIG. 2:**
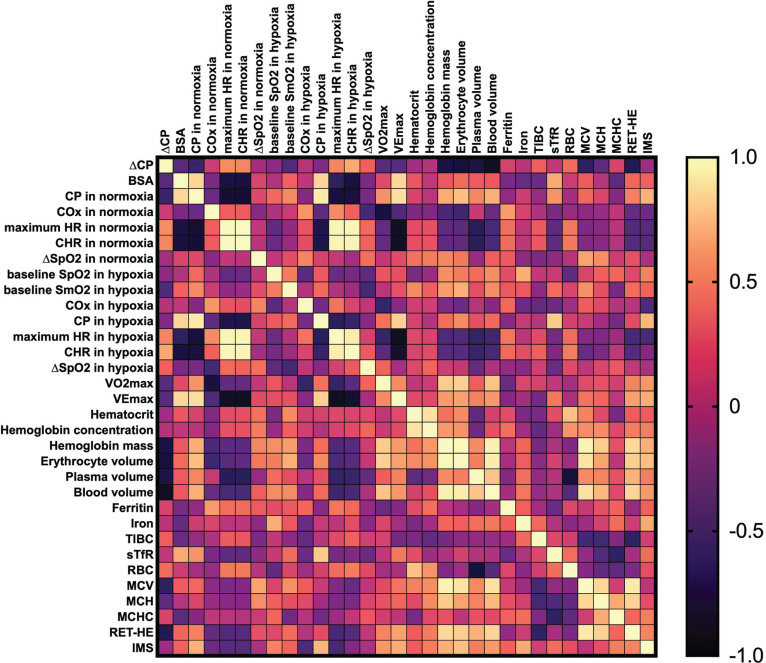
Pearson’s correlation matrix for key analysed variables in females. Abbreviations: ∆CP – differences in critical power between normoxia and hypoxia, BSA – body surface area, CP – critical power, COx – critical oxygenation, SpO_2_ – peripheral capillary oxygen saturation, SmO_2_ – muscle oxygen saturation in vastus lateralis, HR – heart rate, CHR – critical heart rate, ${\dot{\text{V}}\text{O}}_{2\max}$ – maximum oxygen uptake, VEmax – maximum ventilation, TIBC – total iron-binding capacity, sTfR – soluble transferrin receptor, RBC – red blood cell (count), MCV – mean corpuscular volume, MCH – mean corpuscular haemoglobin, MCHC – mean corpuscular haemoglobin concentration, RET-HE – reticulocyte haemoglobin equivalent, IMS – inspiratory muscle strength.

**FIG. 3:**
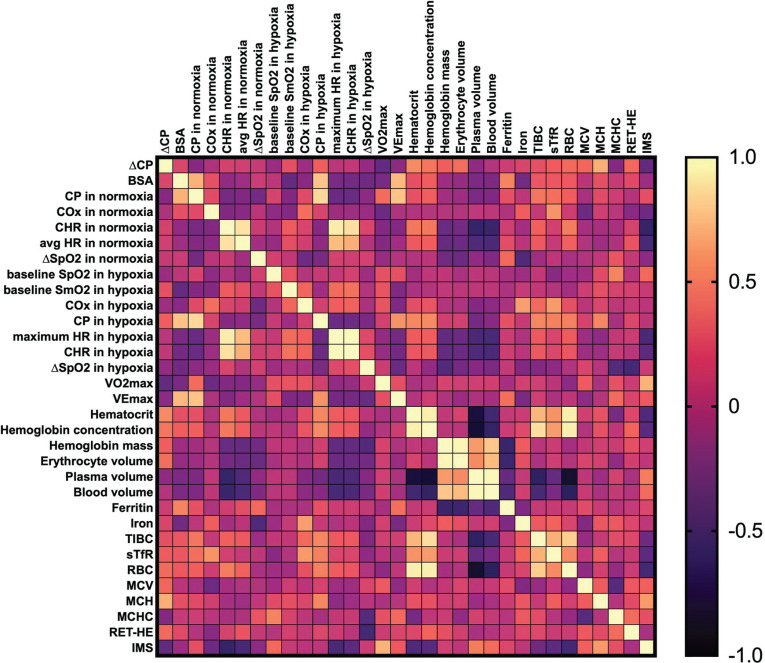
Pearson’s correlation matrix for key analysed variables in males. Abbreviations: ∆CP – differences in critical power between normoxia and hypoxia, BSA – body surface area, CP – critical power, COx – critical oxygenation, SpO_2_ – peripheral capillary oxygen saturation, SmO_2_ – muscle oxygen saturation in vastus lateralis, HR – heart rate, CHR – critical heart rate, ${\dot{\text{V}}\text{O}}_{2\max}$ – maximum oxygen uptake, VEmax – maximum ventilation, TIBC – total iron-binding capacity, sTfR – soluble transferrin receptor, RBC – red blood cell (count), MCV – mean corpuscular volume, MCH – mean corpuscular haemoglobin, MCHC – mean corpuscular haemoglobin concentration, RET-HE – reticulocyte haemoglobin equivalent, IMS – inspiratory muscle strength.

No significant effects were observed for ∆CP correlation with any other variables (p > 0.05). We underline that ∆CP is a percentage change transformed with the log_10_ approach; hence, a lower ∆CP value means a greater decrease in CP between environments (see the Methods section). For example, descriptively, individuals with higher ${\dot{\text{V}}\text{O}}_{2\max}$ and baseline CP in normoxia tend to experience a greater decrease in CP under hypoxic conditions. The correlation matrices are presented as Figures 1–3.

Multiple linear regression was used to identify the most important predictors for ∆CP. The analysis revealed that the best-performing model (F_(3, 22)_ = 7.034, R = 0.708, R^2^ = 0.501, RMSE = 0.033) included MCH, ${\dot{\text{V}}\text{O}}_{2\max}$, and Hb_mass_, and yielded a statistical significance with a large effect size (p = 0.002, Cohen’s F^2^ = 1.004):


$$\Delta \text{CP}=2.097+(0.044\times \text{MCH})-(0.005\times {\dot{\text{V}}\text{O}}_{2\max})+(0.001\times {\text{Hb}}_{\text{mass}})$$

Notably, the predictors exhibited different significance levels: the p-value for MCH was 0.006, for ${\dot{\text{V}}\text{O}}_{2\max}$ was 0.016, and for Hb_mass_ was 0.850. Although Hb_mass_ was not a significant individual predictor, its inclusion improved the model’s overall explanatory power (∆R^2^ = 0.204), suggesting a potential suppressor effect within the multivariate context.

## DISCUSSION

The differences in CP between normoxia and hypoxia were investigated in relation to fitness level and haematological status in healthy, highly trained endurance athletes. In the combined female and male sample, the decrease in CP in hypoxia was greater in athletes with a higher fitness level, as identified by ${\dot{\text{V}}\text{O}}_{2\max}$ and baseline CP in normoxia. Notably different outcomes were observed for separate female and male samples. Crucially, all haematological indices assessed with the CO rebreathing method were strongly or very strongly correlated with ∆CP, but only in females. Moreover, ∆CP was strongly correlated with MCV and RET-HE. In males, ∆CP was moderately correlated with haematocrit and strongly correlated with MCH. The presented regression model for ∆CP was statistically significant, exhibited a large effect size, and included MCH, ${\dot{\text{V}}\text{O}}_{2\max}$, and Hb_mass_.

Extensive research has already been conducted on hypoxia-induced changes in ${\dot{\text{V}}\text{O}}_{2\max}$ across populations with markedly different fitness levels. The current evidence appears consistent, with multiple studies reporting a greater reduction of ${\dot{\text{V}}\text{O}}_{2\max}$ in highly trained individuals compared to their untrained counterparts under hypoxic conditions [12, 31]. However, research focusing on already trained populations remains limited. As this study examined a highly trained cohort, identifying such an association in the investigated sample represents a novel contribution that may be useful for practitioners working in high-performance environments. Similar outcomes in skimountaineers and higher ${\dot{\text{V}}\text{O}}_{2\max}$ reduction in hypoxia in elites compared to recreational athletes were reported [32]. Notably, ${\dot{\text{V}}\text{O}}_{2\max}$ does not equal specific performance. In our study, athletes with higher baseline CP in normoxia also exhibited larger CP drops in hypoxia, indicating practical implications of the present results. Although altitude training has traditionally been associated with high-performance environments, it has increasingly gained attention among the amateur athletic population. Our findings suggest that the translation of established hypoxia training guidelines from elite to amateur athletes requires considerable caution.

To our best knowledge, no studies have investigated changes in performance between different oxygen availability conditions in already trained athletes with proven adequate haematological status. Multiple studies have shown that in untrained individuals, oxygen consumption primarily determines ${\dot{\text{V}}\text{O}}_{2\max}$, while in endurance-trained athletes, the main limiting factor is blood oxygen supply [33, 34]. In normoxia, endurance performance exhibited by ${\dot{\text{V}}\text{O}}_{2\max}$ typically depends on cardiac output and haemoglobin concentration. In hypoxia, the oxygen diffusion rate in the lungs and skeletal muscles becomes the limiting factor [35]. In this context, both haemoglobin concentration (associated with oxygen transport capacity) and mass (associated with both oxygen transport capacity and erythrocyte volume) have significant effects on endurance performance exhibited by ${\dot{\text{V}}\text{O}}_{2\max}$ [34]. Consequently, even mild anaemia may significantly affect physiological responses to hypoxia, with anaemic individuals typically exhibiting a greater decline in ${\dot{\text{V}}\text{O}}_{2\max}$ at altitude [36, 37]. However, there is no evidence whether “more is better”, and whether a superior haematological status mitigates performance drop at altitude in populations with an already sufficient blood profile. Interestingly, oxygen transport at altitude seems unaffected by haemoglobin concentrations as low as the 10^th^ percentile of the population mean [36]. In the present study, only a few significant associations were found between the indices obtained from venous blood sampling and ∆CP. Our findings do not allow us to conclude whether, once haematological status assessed by biochemical assays is adequate, higher values of relevant indices help mitigate the magnitude of performance decline in hypoxia. However, in females, ∆CP was associated with all analysed haematological variables obtained with the CO rebreathing method. This finding aligns with prior research showing that 1) haematological status plays a more prominent role in female exercise performance, likely due to well-established sex disparities [38]; 2) the CO rebreathing method offers unique physiological insights and should be considered a preferred approach for assessing haematological status in highly trained athletes [39, 40].

In the present study, neither ∆SpO_2_ nor ∆SmO_2_, was significantly associated with ∆CP. Initially, we speculated that local oxygenation kinetics was not related to ∆CP, as typically SmO_2_ is similar during exercise in normoxic and hypoxic conditions [41–43]. Despite the absence of significant differences in SmO_2_ across different ambient oxygen concentrations during the tests, performance declined under hypoxia [18]. This suggests that factors beyond local muscle oxygenation may contribute to the observed impairment. One potential explanation involves the central nervous system, which is highly sensitive to even mild reductions in arterial oxygen content [44]. Cerebral oxygen delivery may remain compromised in hypoxia, leading to the earlier onset of central fatigue and reduced motor drive [45]. Importantly, multiple studies have mechanistically linked the magnitude of ${\dot{\text{V}}\text{O}}_{2\max}$ decrease in hypoxia with a drop in SpO_2_ [12, 31, 32]. In our study, we did not examine changes in ${\dot{\text{V}}\text{O}}_{2\max}$ but rather focused on changes in CP. The corresponding phenomenon was not observed in the investigated sample, as there was no significant correlation between ∆SpO_2_ in hypoxia and ∆CP. This might be due to the relatively homogeneous fitness status of the studied population, as all participants were highly trained endurance athletes. Nevertheless, the lack of association between ∆CP and ∆SpO_2_ stands in contrast to the available findings that trained populations exhibit significant arterial desaturation under hypoxic conditions, and simultaneously, the decline in endurance performance depends on the rate of arterial desaturation [46, 47]. This discrepancy suggests that in highly trained individuals, the relationship between ∆SpO_2_ and performance decline in hypoxia may be modulated by physiological or compensatory mechanisms, such as enhanced buffering capacity, oxygen delivery, or muscular oxygen extraction. However, mechanistic studies employing highly trained cohorts are warranted to clarify determinants of performance impairment under hypoxic conditions.

Notably, the regression model presented a large effect size (Cohen’s F^2^ = 1.004), and explained 50.1% of variance, with variables exhibiting substantially different individual significance levels. These results underscore the importance of evaluating predictors within multivariate frameworks, as the contribution of individual variables may be masked or enhanced by interrelationships with others. Accordingly, future studies involving larger cohorts are warranted to enable the inclusion of additional predictors without compromising statistical rigor.

### Strengths, limitations, and further research

The novelty and application of a wide range of assessments, performed by experienced staff using validated methods, were the main strengths of this study. Although the sample may be considered small, a unique population of highly trained athletes was investigated. Unfortunately, the sample size forced the combined regression analysis of female and male athletes together. A larger cohort might have allowed for the development of an improved and more accurate model. The physiological indices from cardiopulmonary tests, blood tests, and CO rebreathing were collected from 4 weeks before to 4 weeks after the cycling tests, which remains a notable study limitation. During such a period, these indices may have slightly fluctuated due to typical temporal variations, potentially affecting the consistency of the findings. Moreover, in athletes with very low body fat, BSA may not accurately reflect body composition, potentially affecting the interpretation of normalized physiological values. However, Mosteller’s formula that was applied in our study exhibits a low systematic error (under 1.5%) and low random error (standard deviation of the differences of approximately 2%) [48]. Estimating CP from a single test is often applied; however, employing at least 2 testing efforts would significantly improve the reliability of such measurements [48]. Furthermore, since swimming remains a considerable part of triathlon training, the included triathletes might occasionally be exposed to short hypoxia bouts [49]. Although no significant differences between triathletes and speedskaters were observed, we cannot exclude the possibility that the swimming training might have been a confounding factor. Finally, a cross-sectional study design was applied, and causal inferences should not be drawn.

Our investigation applied acute simulated altitude in a normobaric hypoxia chamber. Altitude training and competition are typically performed in terrestrial hypobaric hypoxia, which induces different physiological responses [50], likely due to differences in barometric pressure [51]. Therefore, further work might employ hypobaric hypoxia. Moreover, the investigated indices might change during a sojourn at altitude [52]; therefore validation of our findings in relation to the degree of acclimatization is warranted. Future research should aim to obtain a larger sample of females and investigate different performance tests, concerning competition-specific performance or durability.

## CONCLUSIONS

A higher fitness level is associated with a greater CP decrease in hypoxia among the homogeneous cohort of highly trained endurance athletes. Our findings are inconclusive on whether, once haematological status assessed by biochemical assays is adequate, higher values of relevant indices mitigate the magnitude of performance decline in hypoxia. However, in females, ∆CP was associated with all analysed haematological variables obtained with the CO rebreathing method. This suggests that haematological status plays a more prominent role in female exercise performance, and the CO rebreathing method should be considered a preferred approach for assessing haematological status in highly trained athletes, especially females. Further research in terrestrial hypobaric hypoxia, considering the degree of acclimatization, seems warranted.
